# Irradiation or temozolomide chemotherapy enhances anti-CD47 treatment of glioblastoma

**DOI:** 10.1177/1753425919876690

**Published:** 2019-09-23

**Authors:** Sharareh Gholamin, Osama A Youssef, Marjan Rafat, Rogelio Esparza, Suzana Kahn, Maryam Shahin, Amato J Giaccia, Edward E Graves, Irving Weissman, Siddhartha Mitra, Samuel H Cheshier

**Affiliations:** 1 Division of Pediatric Neurosurgery, Department of Neurosurgery, Lucile Packard Children’s Hospital, Stanford University School of Medicine, USA; 2 Institute for Stem Cell Biology and Regenerative Medicine and the Stanford Ludwig Cancer Center, Stanford University School of Medicine, USA; 3 Division of Pediatric Neurosurgery, Department of Neurosurgery, Huntsman Cancer Institute, School of Medicine, University of Utah, USA; 4 Department of Radiation Oncology, Stanford University, USA; 5 Department of Pediatrics, Hematology/Oncology/Bone Marrow Transplant Research Laboratories, Children’s Hospital Colorado, University of Colorado, School of Medicine, USA

**Keywords:** Glioblastoma, anti-CD47, irradiation, temozolomide, phagocytosis, macrophages

## Abstract

Irradiation and temozolomide (TMZ) chemotherapy are the current standard treatments for glioblastoma multiforme (GBM), but they are associated with toxicity and limited efficacy. Recently, these standard therapies have been used to enhance immunotherapy against GBM. Immunotherapy using the anti-CD47 (immune checkpoint inhibitor) treatment has shown promise in treating multiple tumor types, including GBM. The goal of this current work was to test whether irradiation or TMZ chemotherapy could enhance anti-CD47 treatment against GBM. Our results showed that irradiation and TMZ each significantly enhanced anti-CD47-mediated phagocytosis of GBM cells *in vitro*. Furthermore, mice engrafted with human GBM that received anti-CD47 combined with focal irradiation or TMZ treatment showed a significant increase in the survival rate compared to those that received a single treatment. The tumor growth in mice that received both anti-CD47 and irradiation was significantly less than that of groups that received either anti-CD47 or focal irradiation. The results from this study may support future use of anti-CD47 treatment in combination with irradiation or chemotherapy to enhance the therapeutic efficacy of GBM treatment.

## Introduction

Glioblastoma multiforme (GBM) is one of the most devastating brain tumors in adults, with an average median survival of 18 mo.^1,2^ The current standard treatment consists of maximal safe resection followed by irradiation and chemotherapy with the alkylating oral agent temozolomide (TMZ). Despite an initial modest response to irradiation and chemotherapy after surgical removal of the tumor, the recurrent tumor demonstrates resistance to irradiation and chemotherapy, resulting in poor clinical outcomes.^1,3^

Recent advancements in immunotherapy have offered new hope for improving GBM outcomes. Modulating the innate immune response against brain tumor cells using anti-CD47 Abs showed promising results in treatment of multiple brain tumor types in preclinical studies, including GBM.^4–7^ CD47 is a ubiquitous protein expressed on the surface of most tumor cells. The interaction of CD47 with signal-regulatory protein alpha (SIRPα) on the macrophage cell membrane leads to inhibition in the macrophage-mediated phagocytosis. *In vitro* and *in vivo* studies showed that blocking CD47-SIRPα interaction by using anti-CD47 mAbs enhanced macrophage-mediated phagocytosis, improved survival, and reduced tumor burden in human GBM engrafted mice.^4^

Previous studies have demonstrated that irradiation and chemotherapy can enhance the effect of immunotherapies against GBM via various mechanisms.^8^ For instance, irradiation and chemotherapy induce apoptosis in highly proliferating cells, making them more available for phagocytosis by APCs (e.g., macrophages, microglia, dendritic cells), which then present tumor Ags to adaptive immune cells. This effect further activates the antitumor immune response.^9^ In spite of growing research about the effect of irradiation and chemotherapy on T cell–mediated immunotherapies such as anti-PD1 and anti-CTLA4 Abs, the effect of irradiation or chemotherapy on innate immune-modulator therapeutics such as CD47 blockade Abs has yet to be fully elucidated.^10,11^

Here, we evaluated whether irradiation or chemotherapy can enhance anti-CD47 treatment of GBM. We demonstrate that irradiation or TMZ chemotherapy enhanced anti-CD47 treatment by increasing macrophage-mediated phagocytosis of tumor cell *in vitro*. We also demonstrate enhanced effect of the combinations *in vivo* as assessed by survival times and tumor sizes in patient-derived orthotopic xenograft (PDX) mouse models of human GBM after treatment with anti-CD47 Ab combined with either irradiation or TMZ chemotherapy.

## Materials and methods

### In vitro phagocytosis assay

*In vitro* phagocytosis assays with human PBMC-derived macrophages and CFSE-labeled DK-MG or LN-299 GBM cells were performed as described previously.^4^ In summary, cells were irradiated with 2 or 8 Gy, or treated with different concentration of TMZ (0, 25, 100, or 500 µM), and incubated for 24 h at 37°C. Cells were then treated with 10 µg/ml humanized anti-CD47 Ab (Hu5F9-G4) or 10 µg/ml IgG control. PBMC-derived macrophages were incubated with treated and untreated GBM cells for 2 h. Cells were collected and analyzed on flow cytometry and FlowJo v10.5 (FlowJo 9.9.4 (Mac), Ashland, OR). Each condition was performed in triplicate.

We normalized the data against anti-CD47 treatment alone. The mean of each condition was divided by the mean of phagocytosis (% of macrophages that had phagocytosed tumors as determined by flow cytometry) of anti-CD47 only treatment.

### Orthotopic xenografts for in vivo analysis

Immunodeficient NSG mice that retain macrophages but lack lymphoid cells were orthotopically injected with GFP–luciferase-expressing SU_GBM44 primary human GBM cells, as previously described.^4^ The intracranial engraftment was confirmed by bioluminescence imaging (BLI) 10 d after injection. Immediately after BLI, the mice were normalized for tumor growth within each planned group and irradiated (*n* = 7 mice per group). To deliver the focal irradiation precisely to the site of injection, the anesthetized mice underwent computed tomography (CT) imaging, and the holes were detected from tumor implantation. Irradiation (10 Gy) was delivered through a 3 mm beam centered on the site of implantation. The tumors were 1–1.5 mm in diameter on the day of irradiation.^12^ We used 10 Gy in the *in vivo* treatment because Leder et al. found 10 Gy was the minimal irradiation dose that delivered the maximal tumor shrinking when GBM-bearing mice were irradiated with different irradiation doses.^13^

For TMZ treatment, mice were treated with 100 mg/kg/d of TMZ for 7 d. Treatment with Hu5F9-G4 or IgG control followed the irradiation or TMZ in the dual-treated group. Monitoring of the tumor growth was performed every week using BLI. “Death” was determined by veterinary approved criteria for terminal morbidity to avoid animal suffering as described.^4^

### Statistics

Statistical analyses were performed using Graph Pad Prism v8 (GraphPad Prsim 7.0b, San Diego, CA). The log-rank (Mantel–Cox) test was used to calculate *P*-values for animal survival analysis. The Mann–Whitney test was used for group comparisons (two-tailed) of tumor growth. Values are shown as the mean ± SEM. Student’s *t*-test (two-tailed) was used for *in vitro* phagocytosis. All animal procedures were approved by the Animal Care and Use Committee of Stanford Medical School.

## Results

### Irradiation enhanced anti-CD47-dependent phagocytosis of GBM in vitro

**Figure 1.**
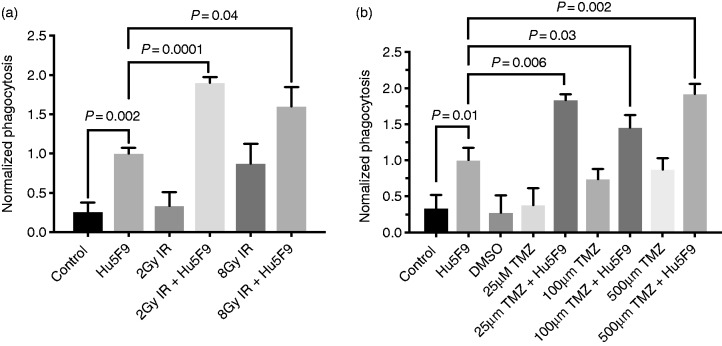
Effect of combining anti-CD47 treatment with irradiation or TMZ chemotherapy on the macrophage-dependent phagocytosis of glioblastoma multiforme (GBM) *in vitro.* (a) GBM tumor cells were treated with Hu5F9-G4 (Hu5F9), 2 or 8 Gy irradiation (IR), or both and subjected to phagocytosis by PBMC-derived macrophages. (b) GBM tumor cells were treated with Hu5F9-G4 (Hu5F9), 0, 25, 100, and 500 µM TMZ, or both and subjected to phagocytosis by PBMC-derived macrophages. Control=non-binding IgG_4_ alone, and DMSO at the same concentration the TMZ was diluted in. Data were normalized against anti-CD47 treatment alone.

To test the effect of combining irradiation with CD47 blockade on GBM, we performed an *in vitro* phagocytosis assay. Human PBMC–derived macrophages and the human GBM cell line were exposed to irradiation with and without a humanized anti-CD47 Ab (Hu5F9-G4). Phagocytosis of tumor cells by macrophages was measured by flow cytometry.^4,14^ As shown in Figure 1a, Hu5F9-G4 alone significantly increased phagocytosis of GBM cells compared to cells that were treated with IgG control. Similarly, GBM cells subjected to irradiation showed significantly higher (only with 8 Gy) phagocytosis compared to control. Interestingly, GBM cells treated with both irradiation (2 or 8 Gy) and Hu5F9-G4 exhibited significantly higher phagocytosis rates when compared to the individual treatments (Figure 1a and Table 1). These results show that irradiation can enhance the phagocytosis of GBM when combined with anti-CD47 *in vitro*.

**Table 1.** *P*-values of all treatment comparisons in the *in vitro* phagocytosis.

Treatment comparison	*P*-value
Irradiation + Hu5F9 treatment	
Hu5F9 vs. control	0.002*
2 Gy IR vs. control	0.587
(2 Gy IR + Hu5F9) vs. 2 Gy IR	0.001*
(2 Gy IR + Hu5F9) vs. Hu5F9	0.0001*
8 Gy IR vs. control	0.035*
(8 Gy IR + Hu5F9) vs. 8 Gy IR	0.024*
(8 Gy IR + Hu5F9) vs. Hu5F9	0.043*
(8 Gy IR + Hu5F9) vs. (2 Gy IR + Hu5F9)	0.16
TMZ + Hu5F9 treatment	
Hu5F9 vs. control	0.010*
25 µM TMZ vs. DMSO	0.616
(25 µM TMZ + Hu5F9) vs. 25 µM TMZ	0.005*
(25 µM TMZ + Hu5F9) vs. Hu5F9	0.006*
100 µM TMZ vs. DMSO	0.059
(100 µM TMZ + Hu5F9) vs. 25 µM TMZ	0.033*
(100 µM TMZ + Hu5F9) vs. Hu5F9	0.031*
500 µM TMZ vs. DMSO	0.030*
(500 µM TMZ + Hu5F9) vs. 25 µM TMZ	0.002*
(500 µM TMZ + Hu5F9) vs. Hu5F9	0.002*

Control = IgG.

*P ≤ 0.05.

HU5F9: Hu5F9-G4; IR: irradiation; TMZ: temozolomide.

### TMZ chemotherapy enhanced anti-CD47-dependent phagocytosis of GBM in vitro

We then tested the effect of combining TMZ chemotherapy with anti-CD47 Ab on tumor phagocytosis. We treated human GBM cells with different concentrations of TMZ with and without Hu5F9-G4. Figure 1b shows that Hu5F9-G4 alone significantly enhanced tumor cell phagocytosis. Only a high concentration of TMZ (500 µM) significantly enhanced the phagocytosis of GBM when administrated alone (*P* ≤ 0.05). However, treating the cells with both TMZ and Hu5F9-G4 significantly improved GBM phagocytosis more than Hu5F9-G4 alone (Figure 1b and Table 1, also see Supplemental Figure). These data showed the positive effect of TMZ on anti-CD47-mediated phagocytosis of GBM *in vitro*.

### Combining irradiation and anti-CD47 inhibited GBM tumor growth and increased survival rate

Finding that combining irradiation with anti-CD47 Ab increased macrophage-mediated phagocytosis of tumor cells *in vitro* led us to hypothesize that irradiation along with anti-CD47 Ab would be more effective in the treatment of intracranial human GBM in our PDX mice models than either treatment alone.

**Figure 2.**
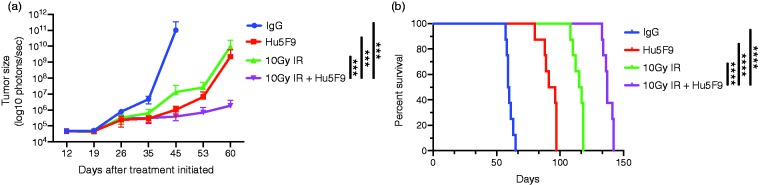
Effect of combining anti-CD47 with irradiation on GBM-bearing mice. (a) Bioluminescence measurements (photons/sec) of GBM and (b) survival rate analysis of PDX mice treated with Hu5F9-G4 (Hu5F9), 10 Gy IR, or both. ****P < 0.0001; ***P < 0.0002.

To test the effect of combining irradiation with Hu5F9-G4 on GBM tumor *in vivo*, we compared the tumor size (as indicated by luciferase imaging intensities) in mice implanted with a GFP-luciferase-expressing human GBM and then treated with Hu5F9-G4 alone, irradiation alone, both, or IgG control. The results in Figure 2a show a significant inhibition in the tumor growth of mice receiving Hu5F9-G4 or irradiation alone compared to controls. However, mice receiving the combination of irradiation and Hu5F9-G4 showed a significant inhibition in their tumor growth compared to mice receiving either Hu5F9-G4 or irradiation only.

To test whether the combination of irradiation and Hu5F9-G4 would further extend survival, we compared the survival of the PDX mice treated with Hu5F9-G4 alone, irradiation alone, or both with untreated controls. Figure 2b shows that Hu5F9-G4 treatment alone extended the median survival of the mice to 93.5 d compared to the control group (59.5 d). Irradiation alone could extend the median survival of the mice to 116 d. The combination of irradiation and Hu5F9-G4 was the most effective, leading to the greatest survival, with a median survival time of 137 d after treatment.

### Combining TMZ chemotherapy and anti-CD47 increased survival rate

**Figure 3.**
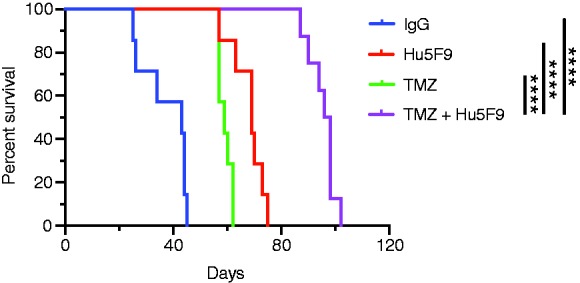
Effect of combining anti-CD47 with TMZ chemotherapy on GBM-bearing mice. Survival rate analysis of PDX mice treated with Hu5F9-G4 (Hu5F9), TMZ, or both. ****P < 0.0001.

As described above, TMZ treatment (alone or in combination with anti-CD47 Ab) showed a significant enhancement in the phagocytosis *in vitro*. We then tested the effect of this combination on GBM *in vivo*. Human GBM PDX mice were treated with Hu5F9-G4 alone, TMZ alone, or both. The results in Figure 3 show a significant progressive improvement in the survival of mice after treating with TMZ, Hu5F9-G4, and a combination of TMZ and Hu5F9-G4, with median survival times of 59, 69, and 97 d, respectively. These data indicate that the TMZ and anti-CD47 Ab combination also enhances survival of human GBM–bearing mice.

## Discussion

The current standard of care for GBM includes maximal resection followed by irradiation and TMZ chemotherapy. Irradiation directly destroys cancer cells by damaging their DNA via free-radical formation. TMZ chemotherapy damages cancer cells by alkylating DNA. Both treatments result in cell-cycle arrest and apoptosis in cancer cells. However, irradiation and chemotherapy can lead to devastating side effects, and these treatments have only resulted in minimal improvements in GBM patient survival.

Recent advances in immunotherapy have great potential to improve outcomes in GBM.^4,15^ Using the immune system to target cancers is especially promising because immune cells possess the molecular machinery are capable of recognizing and killing cancer cells while leaving normal cells unharmed. Both irradiation and chemotherapy have been shown to increase pro-immunogenic signals on cancer cells that can enhance antitumor immune system responses via immunogenic cell death.^2,8^ These pro-immunogenic signals are termed “damage-associated molecular patterns” (DAMPs).^16^ The normal function of DAMPs is to alert the immune system to remove cells damaged by a multitude of processes such as aging, senescence, trauma, viral infection, and bacterial infection. Elevation of DAMPs on cancer cells can enhance the immune clearance of cancer cells as well.^16^ Elevating DAMPs on tumor cells by irradiation and chemotherapy can be achieved by lower doses of irradiation or chemotherapy than standard treatment,^17,18^ thus offering the possibility of combining immune and standard therapies in order to lower doses of irradiation/chemotherapy without sacrificing efficacy. In our study, lower doses of irradiation and TMZ chemotherapy enhanced anti-CD47-mediated tumor phagocytosis, which is in line with these previous studies.

Thus far, the majority of immunotherapy in combination with standard therapies (irradiation and chemotherapy) studies have focused on how the adaptive immune system cells (e.g., T cells) are enhanced. However, the effect of standard therapies on innate immune system modulators such as anti-CD47 has not been as extensively investigated. Nearly all tumors carry anti-phagocytic “do not eat me” signals such as CD47 on their surface. In recent years, CD47 blockade showed very promising results in treating different tumors, including GBM, in preclinical studies.^4,6,19–23^ Humanized anti-CD47 did not show a significant dose-related toxicity in clinical trials.^24^

**Figure 4.**
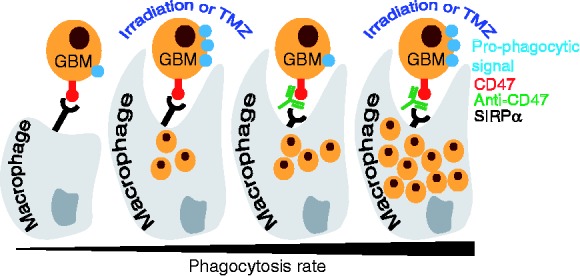
Model of macrophage-dependent phagocytosis after treating GBM cells with anti-CD47, irradiation, TMZ, or a combination of anti-CD47 with irradiation or TMZ. The model shows an enhancement in the macrophage engulfment of GBM cells after combining irradiation or TMZ with anti-CD47 treatment (bar at bottom) via treatment induced increase of pro-phagocytic signals on tumor cells.

Various mechanisms may be involved in the enhanced effect of combining anti-CD47 with irradiation/TMZ. For example, increasing pro-phagocytic signals can tip the balance toward improved anti-CD47 effects. Chao et al. demonstrated that treating tumor cells carrying high levels of pro-phagocytic “eat me” signals with anti-CD47 can improve macrophage-mediated tumor phagocytosis and improve patient outcomes.^25^ It was also shown that increasing pro-phagocytic “eat me” signals via exposure of tumor cells to tumor-targeting mAbs can improve macrophage-mediated tumor phagocytosis.^26^ Given that irradiation and chemotherapy have been shown to increase DAMPs on tumor cells,^27^ and these DAMPs include pro-phagocytic signals involved in the CD47-SIRPα axis (calreticulin, phosphatidylserine, etc.), we hypothesized that combining anti-CD47 immunotherapy with irradiation or TMZ could further improve therapeutic treatment against GBM (Figure 4). Another mechanism of immune system enhancement against tumors in response to irradiation or chemotherapy is the Stimulator of IFN genes (STING) pathway.^28^ STING acts as a sensor of tumor DNA damage caused by irradiation or chemotherapy, prompting the destruction of these tumor cells by the immune system.^28^ STING has been implicated in the activation of macrophages and dendritic cells against tumors in response to irradiation and may be involved in enhancing the phagocytosis of GBM as well.^29^ It should also be noted that CD47 is expressed on a wide range of tissues, and CD47-SIRPα blockade has effects on other immune cells. For instance, inhibition of CD47 has been demonstrated to stimulate potent antitumor effects in other innate immune cells such as neutrophils, dendritic cells, and NK cells,^30^ and these cells are also modulated in response to irradiation/chemotherapy. Thus, a myriad of possibilities of how irradiation/chemotherapy enhances anti-CD47 exist.

The data presented here support our hypotheses that combination of CD47 blockade and irradiation or chemotherapy enhances GBM treatment, as irradiation or TMZ in combination with anti-CD47 increased macrophage-dependent phagocytosis of GBM cells and extended survival of GBM-implanted mice.

A previous study used CD47 blockade in the setting of irradiation mainly as a radioprotectant.^31^ Maxhimer et al. demonstrated that CD47 blockade with either anti-CD47 mAb or CD47 morpholino oligonucleotides could suppress melanoma tumor growth when combined with irradiation. Interestingly, the CD47 blockade in their study reduced irradiation-induced apoptosis in the tumor cells as part of the radioprotective effect of CD47 blockade. Their study also demonstrated an enhanced infiltration of macrophages within tumors treated with irradiation and CD47 blockade. They proposed that the radioprotective effect of CD47 blockade rescued blood-vessel loss from irradiation damage, allowing more immune cells (such as macrophages) to enter the tumor and suppress its growth. Their data are consistent with our previous research demonstrating increased intratumoral macrophages, increased macrophage phagocytosis of tumor cells, and a shift toward a more pro-inflammatory/antitumor/M1 macrophage phenotype in human GBM PDX mice treated with anti-CD47.^4,6,7^ Furthermore, brain-resident microglia (which are embryologically distinct from bone marrow–derived macrophages) can also be stimulated to phagocyte GBM in response to anti-CD47.^7^ Future studies to quantify the amounts of macrophages/microglia within tumors and directly measure *in vivo* tumor phagocytosis in response to anti-CD47 + irradiation/chemotherapy will help to address this issue.

CD47 crosslinking with different mAbs has been demonstrated to induce apoptosis in cancer cells in a manner felt to be similar to CD47 binding another natural ligand, Thrombospondin.^32–34^ The anti-CD47 used in our study, Hu5F9-G4, has been demonstrated to block the interaction between CD47 and SIRPα.^14^ There have been no studies to suggest that Hu5F9-G4 either blocks or mimics CD47–thrombospondin interaction. Furthermore, we and others have shown that Hu5F9-G4 does not cause apoptosis of tumor cells.^4,14^ However, given the result of this study, it would be important to determine whether Hu5G9-G4 can positively or negatively affect the rates of apoptosis in tumor cells in the context of combined treatment with irradiation or chemotherapy.

Combining immunotherapy with irradiation or chemotherapy has different implications in an immunocompetent setting than in an immunodeficient setting. In our study, the immunodeficient mouse model did not reflect the effect of anti-CD47 combined with irradiation/TMZ on the adaptive immune system. Mathios et al. demonstrated that local chemotherapy can enhance anti-PD-1 effect against GBM compared to systemic chemotherapy in PDX mouse models.^35^ Furthermore, the study demonstrated that systemic chemotherapy inhibited T cells, resulting in decreased efficacy. In an immunocompetent setting, systemic TMZ administration will result in lymphopenia. However, one advantage of anti-CD47 is that a main treatment effect is macrophage/microglia-mediated phagocytosis of tumor cells, which may not be altered by TMZ therapy. The macrophages were not affected by the doses of TMZ in our study, given the enhanced activity seen. Specific T-cell responses against tumors can be induced by anti-CD47 treatment alone in an immunocompetent mouse tumor model.^22^ Therefore, the overall response (T-cell + macrophages) may be reduced with systemic TMZ chemotherapy in an immunocompetent model as well as in humans. As with chemotherapy, high doses of irradiation can inhibit T cells, but high doses of irradiation used in our study certainly did not negatively impact the efficacy of anti-CD47 but rather enhanced the effect.

Irradiation or chemotherapy can also enhance tumor removal in combination with immunotherapies. Irradiation and chemotherapy can stimulate T-cell activity by up-regulating DAMPs on tumor cells, making them more “visible” to the adaptive immune system.^36,37^ In a previous mouse melanoma model, combining anti-CD47 with irradiation enhanced tumor shrinkage and survival in an immune intact system, but macrophage-mediated phagocytosis of tumor cells was not studied.^31^ As mentioned above, DAMPs also include pro-phagocytic signals, which make tumor cells more “visible” to macrophages, which is why we suspect the macrophage phagocytosis of tumor cells was enhanced by the combinations. Thus, further studies on the effects of our combination strategy on GBM in an immune intact model are needed to ascertain the relative contributions of innate and adaptive immune cells in this response.

In summary, we have demonstrated that Hu5F9-G4 (anti-CD47) immunotherapy in combination with irradiation or TMZ chemotherapy enhances the eradication of GBM. This response is in part due to the ability of irradiation and TMZ to enhance macrophage-dependent phagocytosis of GBM cells. Clinical trials have already been conducted combining irradiation or chemotherapy with T-cell checkpoint inhibitors such as anti-PD1.^38^ Trials combining Hu5F9-G4 with chemotherapy are underway as well.^24^ Our results can help inform future clinical trials where anti-CD47 is combined with irradiation or chemotherapy regimens and offer the possibility of equal or greater treatment efficacy with lower doses of irradiation and/or chemotherapy.

## Supplemental Material

INI876690 Supplemental Material1 - Supplemental material for Irradiation or temozolomide chemotherapy enhances anti-CD47 treatment of glioblastoma Click here for additional data file. Supplemental material, INI876690 Supplemental Material1 for Irradiation or temozolomide chemotherapy enhances anti-CD47 treatment of glioblastoma by Sharareh Gholamin, Osama A Youssef, Marjan Rafat, Rogelio Esparza, Suzana Kahn, Maryam Shahin, Amato J Giaccia, Edward E Graves, Irving Weissman, Siddhartha Mitra and Samuel H Cheshier in Innate Immunity

INI876690 Supplemental Material2 - Supplemental material for Irradiation or temozolomide chemotherapy enhances anti-CD47 treatment of glioblastoma Click here for additional data file. Supplemental material, INI876690 Supplemental Material2 for Irradiation or temozolomide chemotherapy enhances anti-CD47 treatment of glioblastoma by Sharareh Gholamin, Osama A Youssef, Marjan Rafat, Rogelio Esparza, Suzana Kahn, Maryam Shahin, Amato J Giaccia, Edward E Graves, Irving Weissman, Siddhartha Mitra and Samuel H Cheshier in Innate Immunity

## Authors' Note

Sharareh Gholamin is also affiliated with Biology and Bioengineering Program, California Institute of Technology, USA.
