# Supplementary material for: Irradiation or temozolomide chemotherapy enhances anti-CD47 treatment of glioblastoma
Source: Innate Immun. 2019 Sep 23;26(2):130–7. doi: 10.1177/1753425919876690 (PMC7016411; doi:10.1177/1753425919876690)
Supplement: INI876690 Supplemental Material1 - Supplemental material for Irradiation or temozolomide chemotherapy enhances anti-CD47 treatment of glioblastoma [file INI876690_Supplemental_Material1.pdf]

Supplemental Figure 1. Effect of combining anti-CD47 treatment with irradiation or TMZ chemotherapy on the macrophage-dependent phagocytosis of GBM *in vitro.* (A) GBM tumor cells were treated with Hu5F9-G4 (Hu5F9), 2- or 8-Gy irradiation (IR), or both and subjected to phagocytosis by PBMC-derived macrophages. (B) GBM tumor cells were treated with Hu5F9-G4 (Hu5F9), 0, 25, and 500 μM TMZ, or both and subjected to phagocytosis by PBMC-derived macrophages. Control = non-binding IgG_4 alone, and DMSO at the same concentration the TMZ was diluted in. Data are presented as percentage of macrophages that had phagocytosed tumors as determine by flow cytometry.
