# Supplementary figures and images for: Irradiation or temozolomide chemotherapy enhances anti-CD47 treatment of glioblastoma
Source: Innate Immun. 2019 Sep 23;26(2):130–7. doi: 10.1177/1753425919876690 (PMC7016411; doi:10.1177/1753425919876690)

A)

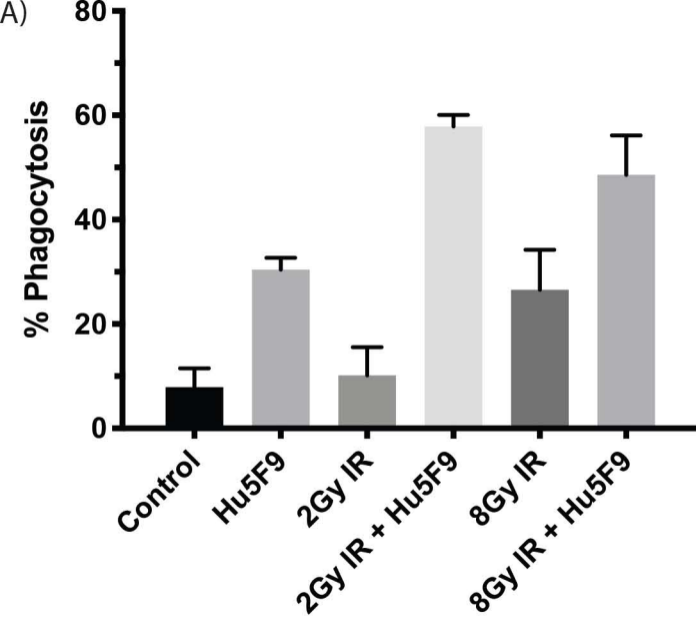

B)

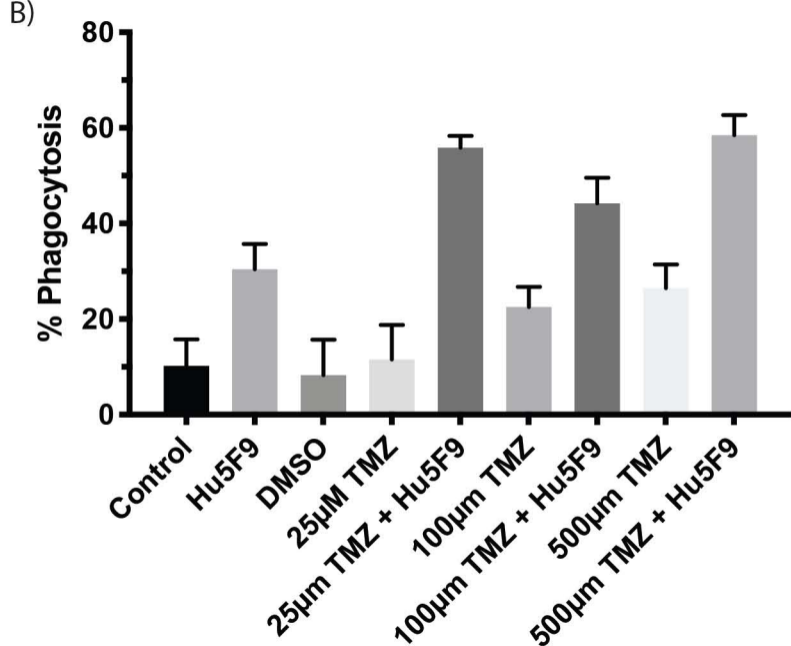

Supplement: INI876690 Supplemental Material2 - Supplemental material for Irradiation or temozolomide chemotherapy enhances anti-CD47 treatment of glioblastoma [file INI876690_Supplemental_Material2.pdf]
